# Metal Cross-Linked Supramolecular Gel Noodles: Structural Insights and Antibacterial Assessment

**DOI:** 10.1021/acs.biomac.4c00300

**Published:** 2024-04-29

**Authors:** Dipankar Ghosh, Sophie M. Coulter, Garry Laverty, Chris Holland, James J. Doutch, Massimo Vassalli, Dave J. Adams

**Affiliations:** † School of Chemistry, University of Glasgow, Glasgow G12 8QQ, U.K.; ‡ School of Pharmacy, Queen’s University Belfast, Medical Biology Centre, 97 Lisburn Road, Belfast BT9 7BL, Northern Ireland, U.K.; § Department of Materials Science and Engineering, Sheffield University, Mappin Street, Sheffield S1 3JD, U.K.; ∥ ISIS Pulsed Neutron and Muon Source, Harwell Science and Innovation Campus, Didcot OX11 0QX, U.K.; ⊥ Centre for the Cellular Microenvironment, University of Glasgow, Glasgow G12 8LT, U.K.

## Abstract

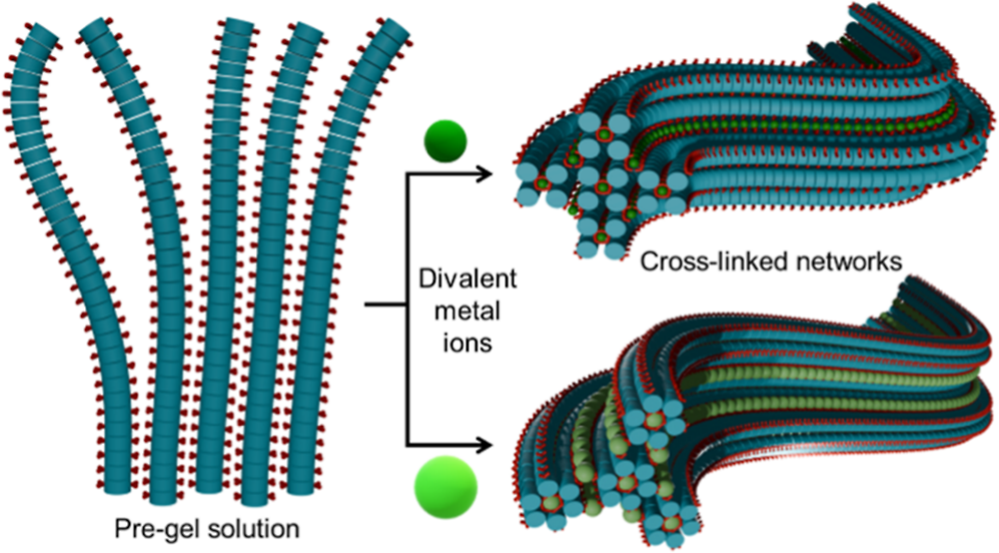

Achieving precise control over gelator alignment and morphology is crucial for crafting tailored materials and supramolecular structures with distinct properties. We successfully aligned the self-assembled micelles formed by a functionalized dipeptide 2NapFF into long 1-D “gel noodles” by cross-linking with divalent metal chlorides. We identify the most effective cross-linker for alignment, enhancing mechanical stability, and imparting functional properties. Our study shows that Group 2 metal ions are particularly suited for creating mechanically robust yet flexible gel noodles because of their ionic and nondirectional bonding with carboxylate groups. In contrast, the covalent nature and high directional bonds of *d*-block metal ions with carboxylates tend to disrupt the self-assembly of 2NapFF. Furthermore, the 2NapFF-Cu noodles demonstrated selective antibacterial activity, indicating that the potent antibacterial property of the copper(II) ion is preserved within the cross-linked system. By merging insights into molecular alignment, gel extrusion processing, and integrating specific functionalities, we illustrate how the versatility of dipeptide-based gels can be utilized in creating next-generation soft materials.

## Introduction

Supramolecular chemistry employs noncovalent interactions to form complex molecular assemblies.^1,2^ Self-assembled supramolecular gels offer exceptional versatility, responding to both environmental cues and exhibiting reversible phase transitions, leading to diverse applications and sparking significant academic and industrial interest.^3−8^ Metal cross-linked supramolecular gels rely on the precise coordination of metal ions with ligands, forming junctions that impart mechanical strength and responsiveness to the gels.^9,10^ Understanding the structure of these gels remains challenging due to their dynamic nature, especially when incorporating metal coordination, which adds complexity and functionality.^9^ The coordination interactions between metal ions and dipeptides dictate the gel’s properties, including mechanical strength and responsiveness to stimuli.^11^ Customizing these properties requires a deep understanding of the gelation process and precise control over supramolecular alignment.^12−14^

**Scheme 1:**
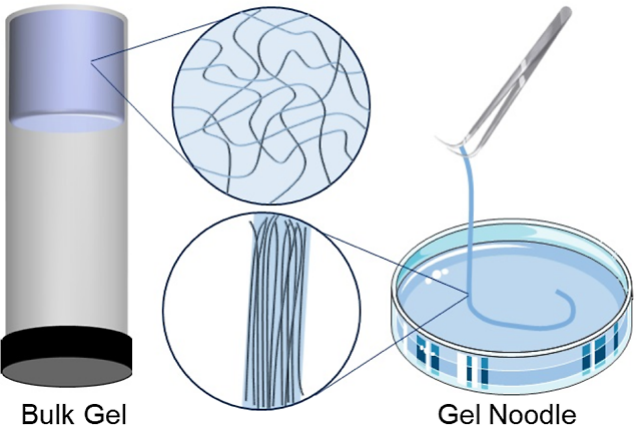
Schematic Illustration of Bulk Gels and Gel Noodles

Efforts to enhance supramolecular alignment have led to innovations in structuring and patterning these gels, transforming them into sophisticated materials capable of maintaining defined forms. For example, gel “noodles” (Scheme 1) have been formed to exploit alignment;^15−17^ with controlled extrusion into metal salt baths enabling the creation of long, well-aligned hydrogel “noodles”, with improved mechanical properties.^18−20^ There are many potential applications for such “noodles” in textiles, biomaterials, microfluidic devices, electronics, sensors, and drug delivery.^18,21−23^

One notable category of supramolecular low molecular weight gels is based on *N*-functionalized dipeptides.^24,25^ These gels, characterized by their biocompatibility and ease of synthesis, find utility in drug delivery, tissue engineering, sensing, and catalysis.^8,26^ These gels can be formed using a range of triggers such as temperature, UV light, pH changes, solvent switches, and metal ions,^27,28^ with the latter representing a useful class of cross-linkers.^9,11^

While previous studies have focused on optimizing pre-gel systems, the role of cross-linking metal ions remains underexplored. Aligning self-assembled nanostructures into macroscopic noodles has been predominantly carried out using CaCl_2_,^16−19,29,30^ with a few examples utilizing cross-linking with HCl or NaCl in a phosphate buffer.^20,21^ Cationic amphiphile cross-linked by divalent anions with high and low charge densities (sulfate and methanedisulfonate, respectively) has also been reported, where the threads obtained with methanedisulfonate were nearly twice as extensible, but their Young’s modulus was almost half that of the threads formed with the sulfate anion.^15^ In this work, we highlight the role of metal ions in shaping supramolecular structures, focusing on a series of divalent metal chlorides. We describe the effect of using different cations on the gels formed, both as bulk gel and as noodles.

## Materials and Methods

All chemicals were sourced from Sigma-Aldrich or Fluorochem Ltd. and utilized as received. Deionized water was employed in all experiments. 2NapFF was synthesized in line with previously documented methods.^31^ The 2NapFF solutions were prepared by stirring 400 mg (0.806 mmol) of 2NapFF, 11.94 mL of deionized water, and 8.06 mL of 0.1 M of NaOH. This blend was stirred continuously for approximately 20 h at ambient temperature (around 20 °C) using a 25 × 8 mm stirrer bar within a 50 mL centrifuge tube at a speed of 750 rpm. The pH level was regulated to 10.5 ± 0.05 using 1.0 M of NaOH.

### Small-Angle Neutron Scattering

The 2NapFF solutions were prepared as described above, utilizing D_2_O and NaOD (1.0 and 0.1 M) for pH adjustment. For gel preparation, an equal volume of freshly prepared 40 mM metal chloride solution in D_2_O was added to the 2NapFF solution in a UV spectrophotometer-grade quartz cuvette (Hellma) with a 2 mm path length. SANS experiments were carried out on the SANS2d instrument at the ISIS Neutron and Muon Source (STFC Rutherford Appleton Laboratory, Didcot, UK) with a source-to-sample and sample-to-detector distance = 8 m and neutrons of the wavelength range of 1.75–14.4 Å to access the *Q* range from 0.0024 to 0.38 Å^–1^. The resulting data were converted to 1D scattering curves (intensity vs *Q*) using Mantid data reduction software.^32^ This process included subtracting the electronic background, normalizing the full detector images, and removing scattering from both the empty cell and D_2_O. The instrument-agnostic data were then analyzed using the SasView software v.4.2.0,^33^ fitting them to the models discussed in the text.

### Rheology

Oscillatory frequency and strain sweep experiments were conducted using an Anton Paar Physica MCR101 rheometer. A 1 mL 2NapFF solution (20 mg/mL, 0.04 mmol, at pH 10.5) was placed in a 7 mL Sterilin vial, to which 1 mL of 40 mM metal chloride (0.04 mmol, 1:1 ratio) or 80 mM HCl (to keep the chloride ion concentration consistent) solution was added. The mixture was gently shaken for approximately 10 s to ensure even mixing and then left undisturbed for 7 days to attain equilibrium. The vial was then positioned on the rheometer and measurements were conducted using a rotating vane geometry with 1 cm width, maintaining a gap distance of 1.8 mm between the geometry and the base of the sample vial. Strain sweeps were also performed at 25 °C, using an angular frequency of 10 rad/s. Frequency sweeps were carried out between 1 and 100 rad/s with a target strain amplitude of 0.2% at a temperature of 25 °C. These measurements were replicated three times for frequency sweeps and twice for strain sweeps, with the resulting values being averaged. Error bars denote the standard deviation among these replicates.

### Preparation of Gel Noodles

20 mL of a 0.5 M freshly prepared metal chloride (or 1 M HCl) solution was poured into a Petri dish (90 mm diameter) and rotated on a spin coater at a speed of 100 rpm. The 2NapFF solution was dispensed using a ProSense syringe pump, fitted with a 10 mL syringe and a 21-gauge needle, at a flow rate of 3 mL/min.

### Cross-Polarized Microscopy

Approximately 2 cm of a freshly prepared gel noodle was positioned on a glass slide. Microscopic images were captured using a Leica DM750 microscope set to 5× magnification. Initially, the white light was calibrated, after which the orientation of the polarizer was adjusted for optimum birefringence. The ImageJ software v.1.54h (National Institutes of Health, Maryland, USA) was utilized to integrate scale bars into these images.^34^

### Nanoindentation

Nanoindentation tests were conducted using a Chiaro nanoindentation instrument (Optics11, The Netherlands), adhering to a standardized procedure.^35^ To maximize the comparability of the data, the same cantilever was used for all experiments, with a radius (*R*) of 3 μm and a stiffness (*k*) of 0.54 N·m^–1^. This nanoindenter was installed on the top of an inverted Zeiss Axiovert 200 M microscope. Roughly 3 cm of a gel noodle was snipped using scissors and placed in a glass Petri dish. A metal washer was positioned on top of the noodle to prevent any movement. Deionized water was then added to submerge the noodles and avert drying. The Petri dish was positioned on the microscope stage, aligning the noodles along the *X*-axis. A minimum of 2 matrix scans were carried out on each noodle, with every matrix scan comprising 25 indentations. The interval between successive indentations in a matrix scan was maintained at 6 μm. For data analysis, the forward segment of the gathered force–displacement (*F*–*z*) curves was scrutinized using customized open-source software.^35^ The contact point was determined through the goodness of the fit algorithm,^35^ transforming *F*–*z* curves into force–indentation (*F*–δ) curves. These *F*–δ curves were then analyzed using the Hertz model, up to a maximum indentation of δ = 0.1*R*, to evaluate the elastic characteristics of the gels.

### Tensile Testing Experiments

The axial stiffness of the noodles (along their length) was gauged through tensile testing experiments using a Zwick Z0.5 TN testing machine (Zwick GmbH & Co., KG, Germany) equipped with a 5 N load cell. Approximately 5 cm of a noodle was cut using scissors, removed from the aqueous bath, and to avoid drying out, the ends were quickly twined around two customized grip holders. These holders were separated by a gauge length of 2 cm, and tests were performed at a strain rate of 0.2 cm/min. The diameter of the thread was determined as the average of at least 20 different measurements along the thread axis from separate micrographs. Both the force exerted and displacement were recorded throughout these tests. The force was then converted to stress through the noodles’ cross-sectional area to analyze the noodles’ tensile strength.

### Bacterial Susceptibility Assay

*Staphylococcus aureus* NCTC 10788 and *Escherichia coli* NCTC 10418 were cultured for 18 h at 37 °C in lysogeny broth and adjusted to an optical density of 0.1 at 550 nm in PBS, equating to 1 × 10^8^ cfu/mL, then further diluted (1 in 50) in the broth. A 1 mL aliquot of the bacterial suspension was subsequently dispensed into each well of a 24-well plate. A 2 cm long gel noodle was cut and washed twice by immersing it in a water bath for 10–15 s to remove any adhered metal salts. The thoroughly washed piece of the gel noodle was added to the 24-well plate with the bacterial suspension. Control wells included bacteria in the broth as a growth control (100% survival), PBS alone as a negative sterility control, and 2NapFF-HCl noodle as an inert hydrogel to investigate the impact of the metal ions on bacterial viability. The inoculated microtiter plates were incubated for 24 h at 37 °C. Following this, 20 μL samples were extracted from each well, serially diluted in PBS from 10^–1^ to 10^–8^, and then plated on lysogeny agar for 24 h at 37 °C for colony quantification using the Miles and Misra technique.^36^ Each experiment was conducted in triplicate, with the results presented as the mean (log_10_ cfu/mL) of the replicates.

## Results and Discussion

**Figure 1:**
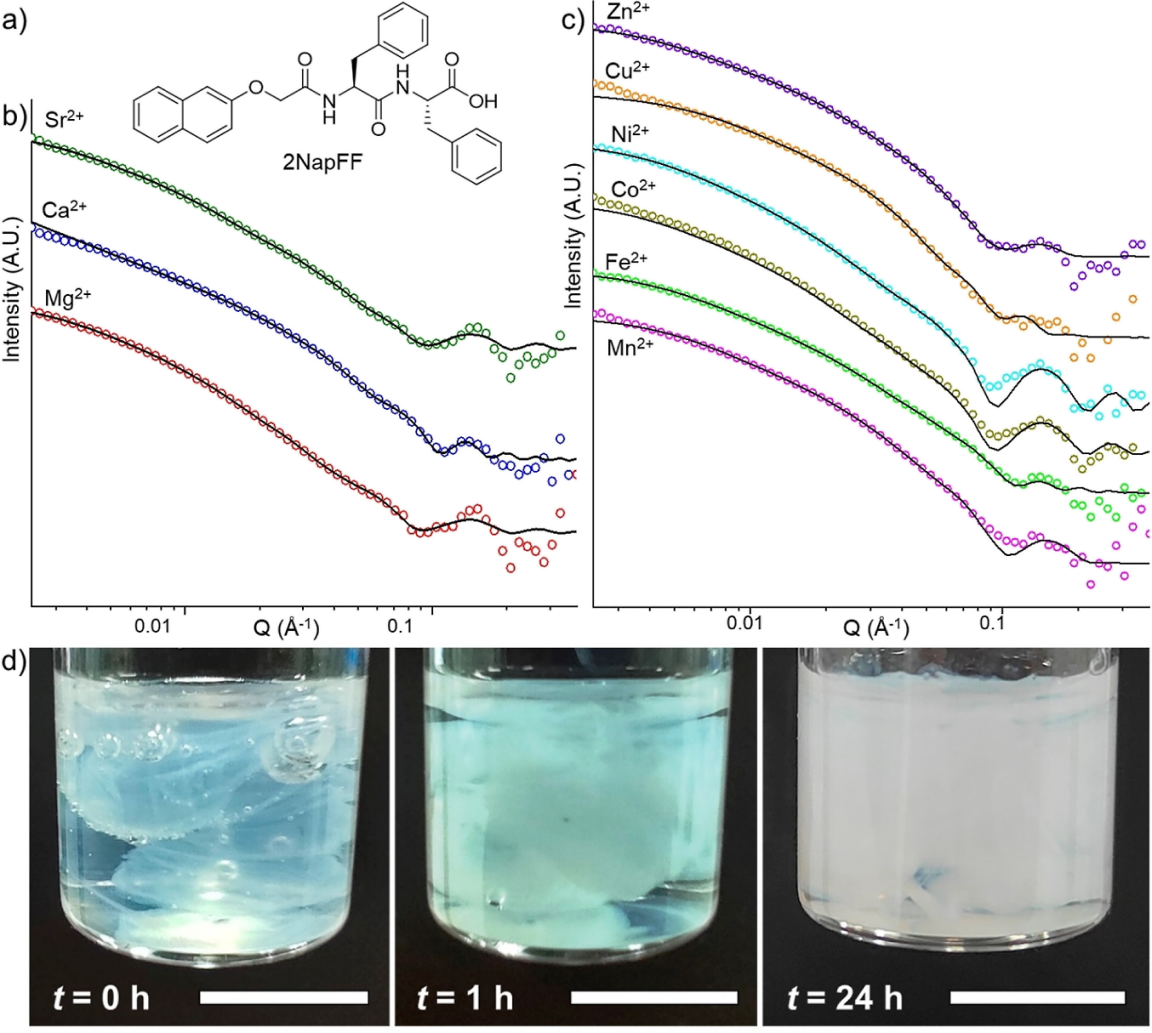
(a) Chemical structure of 2NapFF. (b) SANS data of the 2NapFF bulk gels with Group 2 metal chlorides and (c) with *d*-block metal chlorides. The cations are indicated at the top of each graph. The data are shown as empty circles and the fits as a black line (full fit parameters are listed in Table S1, Supporting Information). The data are offset on the intensity scale for ease of discrimination and the error bars are omitted for clarity. (d) Formation of the cross-linked 2NapFF-Ca gel over time showing the inhomogeneity at an early stage. The scale bars indicate 1 cm.

The *N*-functionalized dipeptide 2NapFF (Figure 1a) self-assembles into long hollow cylinders in aqueous solutions at a high pH.^37,38^ The solutions containing these structures become viscous as the cylinders entangle, leading to spontaneous hydrogel formation in response to various stimuli. Self-assembled gels are formed for example when calcium or magnesium salts are added to a solution of 2NapFF at a high pH.^37^ In this study, our objective is to expand our understanding of how different cross-linkers stabilize and strengthen the gel matrix. Simultaneously, we explore new properties introduced by varying metal ions, specifically focusing on the gel noodle systems. Traditional bulk gels were also studied to understand the structure–property correlations between the bulk gels and noodles.

Considering prior examinations of 2NapFF-calcium bulk gels and noodles,^39^ our initial assessment involved gels derived from 2NapFF in combination with other Group 2 metals, magnesium and strontium chloride. We fixed the 2NapFF concentration at 20 mg/mL, and the pH was adjusted to 10.5 (±0.05 in all cases) using NaOH, based on our previous report that these conditions were suitable for stable bulk gel and noodle formation.^39^ The gel formation strongly depends on the molar ratio of the gelator to the cross-linker,^37^ so we standardized a 1:1 molar ratio of 2NapFF to the metal chlorides for bulk gel analysis. Moving from Mg^2+^ to Ca^2+^ to Sr^2+^ corresponds to an increase in the ionic size, polarizability, and softness, with Mg^2+^ showing the highest binding affinity to carboxylate oxygen, and Sr^2+^ displaying the greatest polarizability.

**Figure 2:**
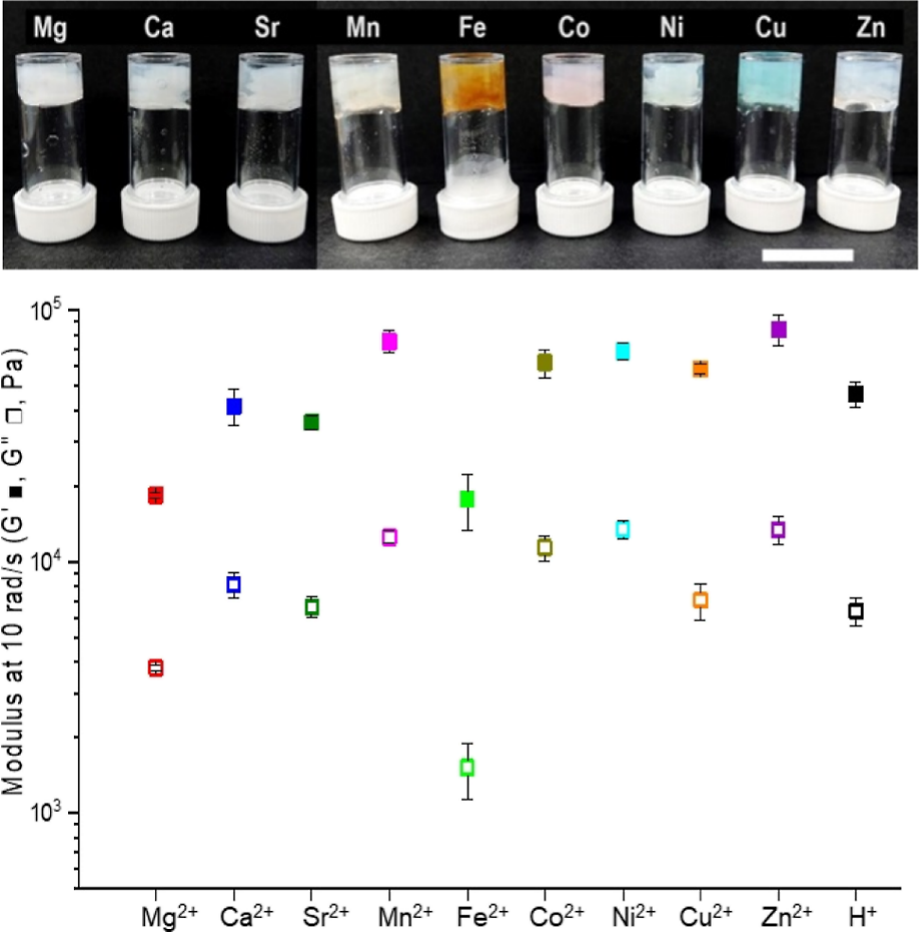
Top: Gels obtained by mixing 2NapFF (20 mg/mL and pH 10.5) and metal chlorides (40 mM), and the scale bar represents 2 cm. The cations are indicated at the top. Bottom: *G*′ and *G*″ of the gels at an angular frequency of 10 rad/s and constant strain of 0.2%, and the cations are given at the *X*-axis.

We also explored gels derived from transition metal chlorides. These *d*-block metal ions form strong coordination covalent bonds with the carboxylate group and introduce unique characteristics like redox activity, color, and magnetism, resulting in hydrogels with intriguing properties.^40,41^ We conducted gelation tests using first-row divalent transition metal chlorides, including MnCl_2_, FeCl_2_, CoCl_2_, NiCl_2_, CuCl_2_, and ZnCl_2_, observing gel formation in all cases (Figure 2).

SANS was performed to identify any structural differences in bulk gels cross-linked with various metal ions. The data, fitted using a flexible elliptical cylinder and hollow cylinder model, indicated complex supramolecular structures likely due to gel inhomogeneity (Figure 1b–d and Table S1). Despite consistent SANS patterns across all gels, slight deviations were observed in the 2NapFF-Fe gel, possibly due to Fe^2+^ oxidation to Fe^3+^, strengthening electrostatic interactions and leading to tighter packing.

Rheological analysis confirmed uniform mechanical properties across different gels (Figures 2 and S1, S2), but specific metal ions had notable effects. Magnesium resulted in softer gels, while weaker gels with FeCl_2_ suggested disrupted networks due to partially oxidized Fe^3+^ (Figure S3). Comparison with pH-triggered gels indicated marginally higher storage modulus (*G*′) for HCl-triggered gels compared to Ca-triggered gels (Figure S1).

We next investigated the process of transforming 2NapFF solutions into 1-D “gel noodles”, which are capable of retaining their original shape upon removal from the mother liquor.^39^ This is significant in the context of shaping supramolecular gels, which can be utilized in developing advanced applications like soft robotics and biointegrated electronics.^42^ Our previous reports have demonstrated that gel noodles showed better nanostructure alignment relative to their bulk counterparts.^19^ Achieving such controlled alignment is critical for enhancing the functionality of the constituent groups in supramolecular systems. Although numerous attempts have been made to assemble highly aligned supramolecular systems, the success is mostly limited to producing short-length fibers or ribbons or films.^15,17,18,29,43^ In contrast, our gel noodles can be extended into significantly longer threads with very high persistence length, allowing for postsynthetic manipulations like folding or weaving.^39^

The gel noodles were prepared by extruding 2NapFF solutions (20 mg/mL, pH 10.5 adjusted with NaOH) from a syringe pump into a rotating 0.5 M solution of various metal chlorides (Figure S4). A high metal ion concentration was maintained to ensure saturation and to test the relative efficiency of each metal cation in stabilizing the noodles. The interaction between the pre-gel solution and the linker led to immediate noodle formation for each salt. The noodles incorporating colored metal chlorides showed a faint color, indicating metal ion incorporation. The nature of the metal ions used made a significant impact. Noodles with Group 2 metal ions demonstrated high tensile integrity and could be lifted with tweezers from the salt bath beyond 1 m in length. In contrast, noodles with *d*-block metal ions were more fragile; for instance, those with Cu^2+^ and Zn^2+^ could only be lifted intact up to 5–10 cm. While for Mn^2+^, Co^2+^, and Ni^2+^, lengths over 30 cm were achievable, but their integrity was inferior to that of noodles with Group 2 metal ions. Noodles formed with 2NapFF-Fe were too brittle for manipulation with tweezers and broke at 1–2 cm in length. Thus, FeCl_2_ produced distinctly different materials from all other salts in both bulk gel and noodles, likely due to the partial oxidation of iron(II) to iron(III), which significantly alters the cross-linking pattern compared to the divalent cations. This shows that divalent metals are more suitable for creating robust noodles with 2NapFF. In this context, we tested the cross-linking of 2NapFF with trivalent aluminum chloride. Although this combination did result in noodle formation, the noodles were too brittle and fractured under minimal stress. Therefore, our study focused on noodles synthesized from 2NapFF and divalent metal chlorides. We also explored HCl-triggered noodle formation by extruding 2NapFF solutions into a 1 M HCl bath, maintaining consistent chloride ion concentration. This comparison will help clarify the role of metal ions in stabilizing the gel noodles.

**Figure 3:**
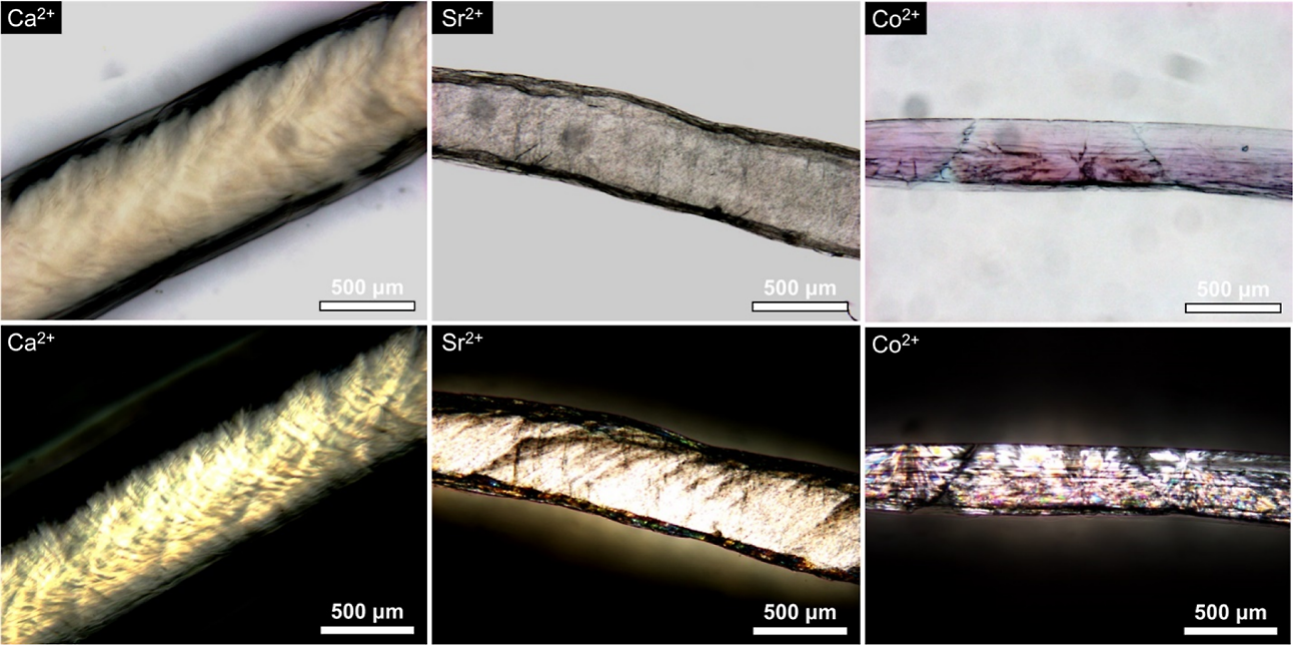
Microscopic (top) and cross-polarized (bottom) images of the gel noodles obtained from 2NapFF and metal chlorides. The cations are indicated at the top left.

Polarization microscopy was employed to analyze fibrillar alignment within the gel noodles. Our results indicate that the noodles were birefringent, indicating alignment and fibrillar features most prominent in noodles containing Group 2 metal ions (Figures 3 and S5), especially with Ca^2+^ and Sr^2+^. In contrast, noodles with *d*-block metal ions displayed less birefringence under polarized light (Figures 3 and S6–S10). Notably, the HCl-triggered noodles showed no birefringence (Figure S11), indicating the lack of anisotropy. This suggests that metal cross-linking is essential to obtain aligned nanostructures.

**Figure 4:**
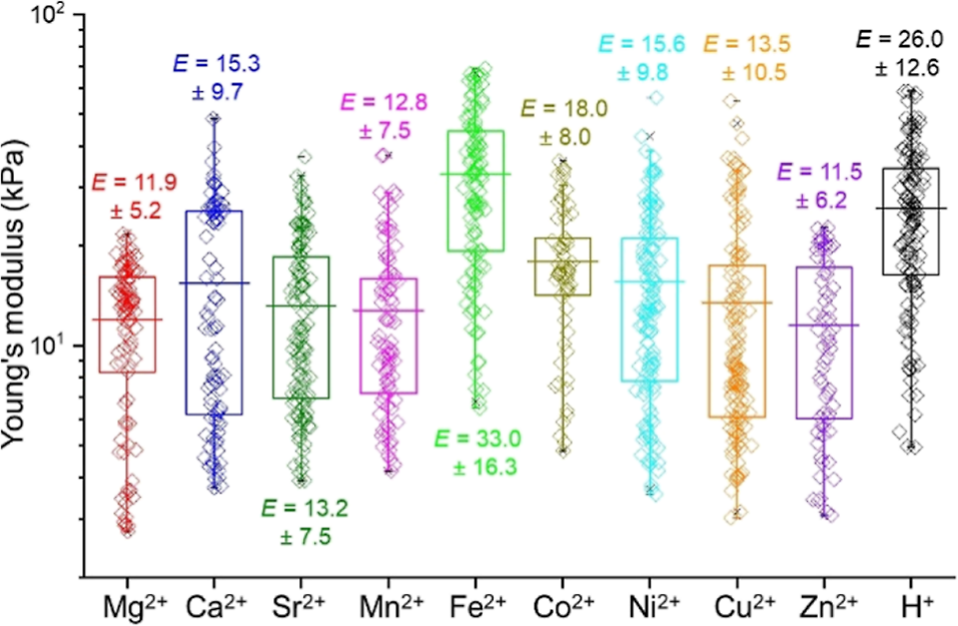
Statistical bar plot of nanoindentation data of gel noodles obtained from 2NapFF and metal chlorides; the cations are shown at the *X*-axis. The average Young’s modulus (*E*) and estimated standard deviation are given (in kPa) adjacent to the bar.

We assessed the elasticity and stiffness of the noodles produced with various metal chlorides. Due to their 1-D shape, traditional bulk rheological measurements were not possible. Instead, nanoindentation was employed to quantify their local mechanical properties at the micron scale.^35,44^ Data from 25 indentations, taken from two distinct regions on each of three different noodles (total of six matrix scans), were compiled. The experiments performed on the noodles formed with Group 2 metal ions revealed that 2NapFF-Ca noodles had slightly higher average Young’s moduli (*E*) compared to 2NapFF-Mg and -Sr noodles, consistent with rheological trends observed in bulk gels (Figure 4 and Tables S2 and S3). For the *d*-block systems, Young’s moduli of these noodles were comparable to the 2NapFF-Ca noodles, except for 2NapFF-Fe noodles. 2NapFF-Fe noodles were unexpectedly stiffer than those formed with other ions (****p < 0.0001, Table S3), contrasting with rheological findings where 2NapFF-Fe gels were the weakest. Such discrepancies are not surprising given the fundamental differences between the bulk gel and noodle systems, where the network in noodles is formed in a more confined space compared to the larger volume of bulk gels. Factors like gelator weight percentage, gelator-to-metal ratio, and the presence of additional ions can significantly influence mechanical properties.^37^ Noodles with various metal ions exhibited Young’s moduli that were similar to each other, consistent with the trends observed in the rheological study. The mechanical strength of 2NapFF-HCl noodles was higher than that of the metal cross-linked noodles (***p < 0.001, Table S3), except for 2NapFF-Fe, presumably due to a more entangled network benefiting from less anisotropy; self-assembled structures and network differences for gels formed from 2NapFF using Ca^2+^ and acids have been described previously.^45^

**Figure 5:**
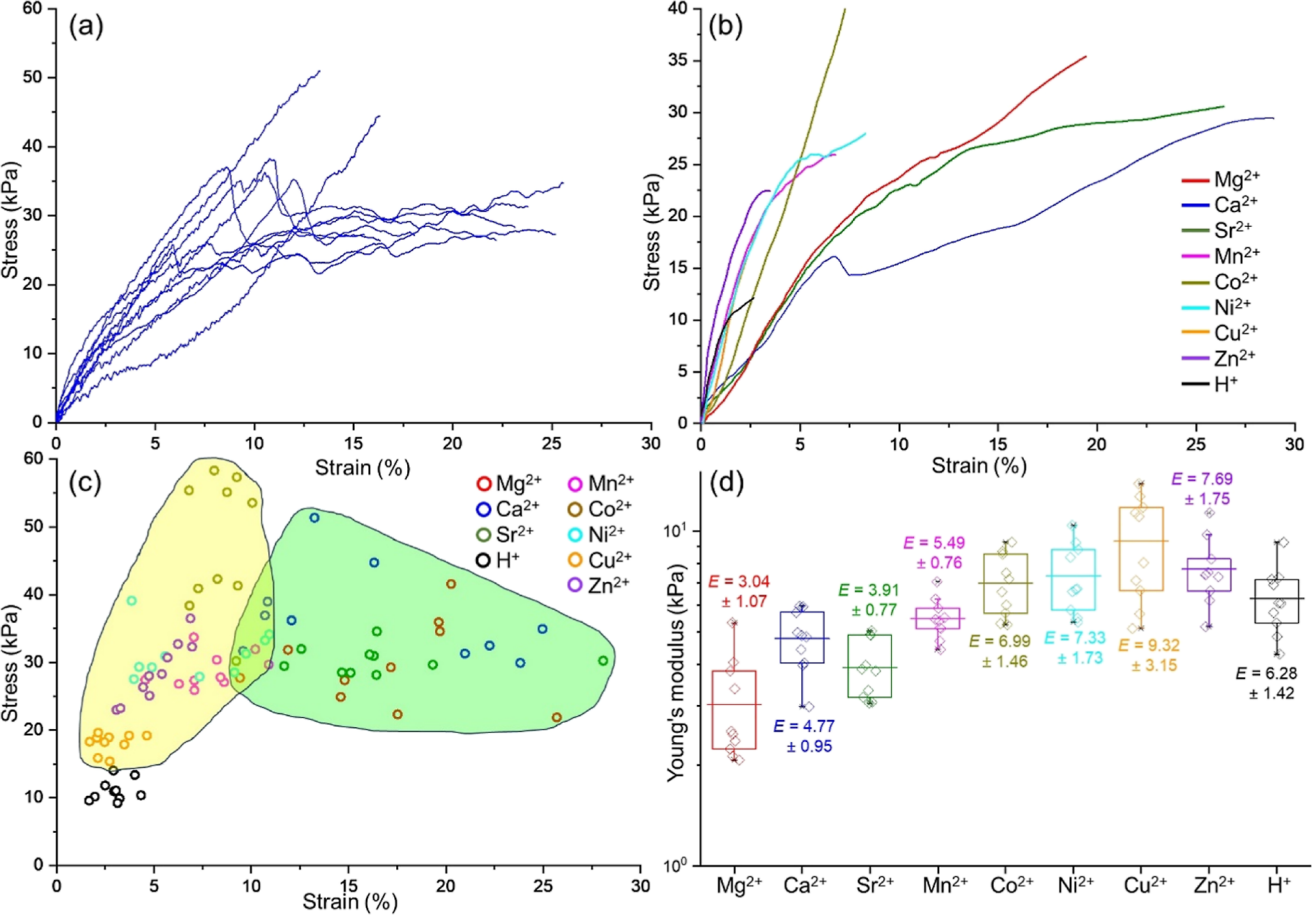
(a) Tensile strength of 10 different 2NapFF-CaCl_2_ noodles showing the distribution of stress and strains at the rupture. (b) Comparative tensile strength of noodles prepared with various metal chlorides, showing one representative data for each cross-linking ion. (c) Rupture point of the gel noodles with different metal ions; the green and yellow zones indicate the noodles with Group 2 and *d*-block metal chlorides, respectively. (d) Young’s modulus (*E*) calculated from the slope of the elastic region of the stress–strain curves.

We further investigated the mechanical properties of gel noodles by assessing their axial stiffness through tensile testing experiments. Each noodle was aligned vertically and stretched at a rate of 2 mm/min until rupture. We tested at least 10 noodles per metal ion and observed the force required for rupture (Figures 5a and S12). The resulting stress–strain curves indicated that noodles with Group 2 metal ions generally had higher ultimate tensile strength than those with *d*-block metal ions, except for Co^2+^ (Figure 5b,c). This finding is in line with the nanoindentation data, where 2NapFF-Co noodles were slightly stiffer than 2NapFF-Ca (ns, *p* > 0.05, Table S3). We also calculated Young’s modulus (*E*) by analyzing the gradient in the elastic region of the stress–strain curves (Figure 5d). The *d*-block metal ions exhibited a higher *E* in comparison to the Group 2 metal ions, as evidenced by the steeper curves associated with these noodles.

The 2NapFF-Fe noodles presented a challenge during analysis due to their brittleness, breaking upon mounting on the tensile tester. This brittleness, contrasting with their micron-level strength, suggests either a loss of flexibility along their length, potentially due to the presence of trivalent Fe^3+^ ions from aerial oxidation of Fe^2+^, or the presence of defects along the noodle that act as stress concentrators because of flow instabilities during formation. Noodles with other *d*-block metal ions like Mn^2+^ and Ni^2+^ had slightly lower ultimate tensile strength than 2NapFF-Ca noodles, whereas Cu^2+^ and Zn^2+^ were significantly weaker. The pH-triggered 2NapFF-HCl noodles exhibited significantly lower tensile properties compared to metal cross-linked noodles (Figure 5c), which can be correlated with the lack of anisotropy observed in cross-polarized images (Figure S11). Since the gel fibrils are aligned along the length of the noodle, the same direction in which the tensile force was applied, noodles with a higher degree of anisotropy displayed greater tensile strength and resistance to deformation in that direction.

The differences in the tensile integrity of the noodles were reflected in the ultimate strain to failure. Group 2 metal noodles could endure 25–30% strain before breaking, indicating high extensibility (Figure 5b,c). This is desirable for applications in stretchable electronics, biomaterials, and soft robotics.^42^ In contrast, most *d*-block metal noodles fractured at just 10% strain, revealing their brittleness (Figures 5b,c and S12). Cu and Zn noodles, enduring only about 5% strain, are unsuitable for stretchable gels. The strain resistance of pH-triggered 2NapFF-HCl noodles was lower than any metal cross-linked noodles (Figure 5c). Thus, the impact of different triggers on mechanical properties was more prominent in noodles than in bulk gels. Altering the trigger enabled fine-tuning of the alignment and mechanical characteristics of the noodles, thereby facilitating the creation of sophisticated soft materials—a feat less attainable with bulk gels.

The distinct tensile properties of the gel noodles of Group 2 and first-row transition metals were correlated to the nature of the metal–carboxylate bonds. Group 2 metal ions form ionic bonds with little covalency and bond directionality, while first-row transition metals create stronger, more directional, and covalent bonds due to effective overlapping with the d-orbitals of the metal ions. This is particularly evident with copper(II) and zinc(II) ions, which form strong, directional bonds, as demonstrated by their prevalence in crystalline metal–organic frameworks, coordination complexes, and polymers.^46,47^ As a result, noodles with these ions exhibited the lowest tensile strength and strain resistance. The nature of the metal chloride (the counteranion in the metal salt) also differs, being more ionic and labile for Group 2 ions, whereas for transition metals, the metal–chloride bonds are more covalent and the chloride ion often acts as a bridging ligand to multiple metal centers, leading to more complex bonding scenarios.

Our findings suggest that Group 2 ions, with their higher ionic nature and less bond directionality, are more suitable for applications requiring increased tensile strength. The strong covalent nature and high bond directionality of the cross-linker in transition metal salts can disrupt the natural self-assembly process of 2NapFF. For instance, with ionic Ca^2+^ ions, 2NapFF forms long 1-D fibrils, as seen in polarized microscopy images, which further intertwine to create robust gel noodles. Conversely, fragmented fibrils were observed in the transition metal noodles, potentially leading to lower strain resistance. Among Group 2 metals, Mg^2+^ has the smallest size, the highest charge density, and the greatest covalency; and among the *d*-block metals, Cu^2+^ and Zn^2+^ ions exhibit the same characteristics, correlating with their weaker mechanical properties. This extends to noodles from trivalent metal ions like Al^3+^ and Fe^2+^ getting oxidized to Fe^3+^, which are strongly covalent and thus have lower mechanical properties.

While Group 2 metal ions enhance mechanical properties, transition metals offer versatility in functionalization such as electrical, thermal, redox, and catalytic properties. However, whether these functionalities can be retained in a gel noodle system remains unexplored. Therefore, we decided to further investigate a potential functional property of these gel noodles. The development of effective antimicrobial agents from biocompatible materials is a significant challenge in materials science and pharmaceuticals.^48,49^ In this regard, peptide-based supramolecular gels, known for their biocompatibility, biodegradability, and low toxicity, have become prominent, especially for their antibacterial properties.^50,51^ The tunable mechanical strength, porosity, and release kinetics of the gels make them ideal for medical applications, allowing for the controlled release of antibacterial agents.^52,53^ A key advancement in this field is the use of metal ion-incorporated hydrogels, which can lead to the inherent antibacterial activity of the gels.^54^

We studied the antibacterial potential of metal cross-linked 2NapFF gel noodles by conducting bacterial susceptibility assays against Gram-positive *S. aureus* (NCTC 10788) and Gram-negative *E. coli* (NCTC 10418). Bacterial viability reduction was measured using a colony counting method, and a 2NapFF-HCl gel noodle was used as a control to determine the impact of metal ions on bacterial viability. Experiments were performed in triplicates, and results were assessed on 24 h old gels to negate aging effects.

**Figure 6:**
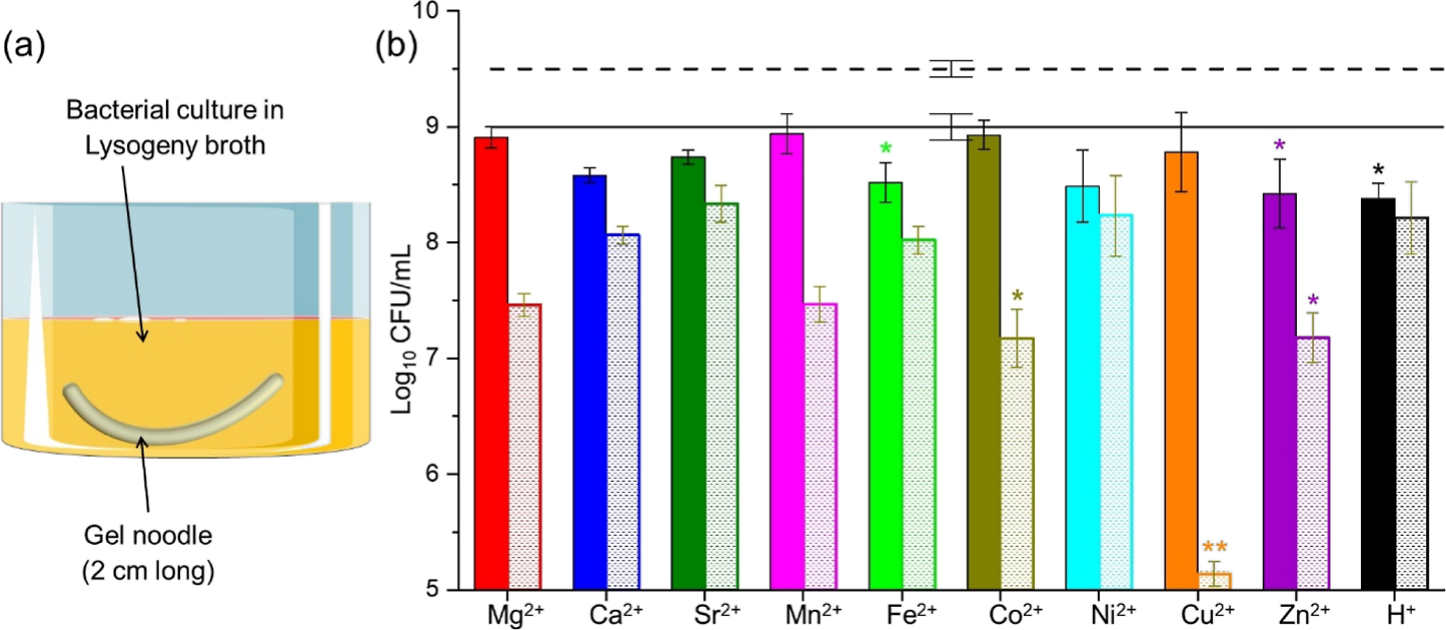
(a) Illustration of bacterial culture with gel noodles. (b) Bacterial susceptibility assays for *E. coli* NCTC 10418 (solid filled) and *S. aureus* NCTC 10788 (dashed pattern). Results displayed as bacterial counts in log_10_ colony forming units per mL (log_10_ cfu per mL). The *X*-axis shows the cations cross-linking the noodles, and the solid and dashed lines on the top show the negative growth control (no noodles added) for *E. coli* and *S. aureus*, respectively. The individual differences between each gel noodle and the negative growth control were identified using the Kruskal–Wallis test, followed by Dunn’s multiple comparison test (*p ≤ 0.05 difference, **p < 0.01 difference, all others are not significant).

Initially, the minimum inhibitory concentration for nine metal chloride solutions was determined, showing normal bacterial growth at 0.05 M for Group 2 metal chlorides and 0.0125 M for transition metal chloride salts. In the assays, 2 cm long noodles of each metal ion, including the HCl noodle, were cultured with bacteria (Figure 6a). The bacterial growth was quantified, with fewer colonies indicating greater antibacterial activity. For *E. coli*, no significant antibacterial activity was observed across all gel noodles (Figure 6b). However, for *S. aureus*, a reduction in colonies was noted with all metal-2NapFF noodles and the HCl noodle, suggesting mild antibacterial activity of the dipeptide against *S. aureus* (Figure 6b). The 2NapFF-Cu noodle demonstrated a significant reduction in *S. aureus* growth with at least a 4-log reduction. At least a 3-log reduction is generally accepted to demonstrate clinical relevance.^55,56^ This shows that incorporating the copper(II) ion into the gel noodle imparts inherent antibacterial properties, primarily due to the strong antibacterial activity of the copper(II) ion.^57,58^ This activity against *S. aureus*, but not *E. coli*, could be due to Gram-negative microorganisms’ tolerance to reactive species, e.g., the copper(II)’s protein efflux system of *E. coli*,^59^ often requiring higher concentrations or prolonged exposure for effective eradication. The study reveals that the functional feature of a metal ion can be preserved in gel noodles. Combining these properties with the shaping of a hydrogelator will enable the fabrication of bioactive soft materials with innovative properties.

## Conclusions

In summary, this study highlights the role of metal ions in stabilizing cross-linked dipeptide-based supramolecular gels. Mixing divalent metal chlorides with 2NapFF pre-gel solution results in the formation of cross-linked gels, which can be shaped into either bulk gels or 1-D gel noodles. The bulk gels are primarily formed by the self-assembly of 2NapFF, with the metal ions cross-linking at the carboxylate ends, resulting in mostly consistent packing and mechanical stiffness. However, the mechanical properties of the gel noodles, as determined by nanoindentation and tensile testing, vary significantly with the type of metal ions used. Noodles incorporating Group 2 metal ions exhibit higher strain resistance, but brittle noodles are formed with *d*-block metal ions. This enhancement is attributed to the long 1-D fibrils observed in the cross-polarized images, which intertwine to form flexible noodles. The analysis of mechanical properties and the nature of the metal ion indicates that the ionic nature of the metal–carboxylate bond is favorable for forming robust yet flexible gel noodles. Thus, Group 2 ions offered mechanical stability to the noodles, whereas transition metal ions, particularly copper, endow the material with significant antibacterial activity against *S. aureus*. The unique combination of these gels’ biocompatibility, biodegradability, and tunable properties, along with their functionalization through metal cross-linking, opens exciting possibilities for the development of sophisticated soft biomaterials and biointegrated devices.

## Supporting Information Available

Additional SANS fitting parameters, rheology data, cross-polarized images, and tensile testing curves (PDF)

## Abbreviations

2NapFF: (2-(naphthalen-2-yloxy)acetyl)-l-phenylalanyl-l-phenylalanine
NaOH: sodium hydroxide
HCl: hydrochloric acid
D_2_O: deuterium oxide
NaOD: sodium deuteroxide
rpm: revolutions per minute
SANS: small-angle neutron scattering
UV: ultraviolet
1-D: one-dimensional
ns: not significant
NCTC: National Collection of Type Cultures
PBS: phosphate-buffered saline
cfu: colony-forming unit
