# Supplementary material for: Metal Cross-Linked Supramolecular Gel Noodles: Structural Insights and Antibacterial Assessment
Source: Biomacromolecules. 2024 Apr 29;25(5):3169–77. doi: 10.1021/acs.biomac.4c00300 (PMC11094724; doi:10.1021/acs.biomac.4c00300)
Supplement: Supplementary file 1 — bm4c00300_si_001.pdf [file bm4c00300_si_001.pdf]

Supporting Information

Metal Cross-Linked Supramolecular Gel Noodles: Structural Insights and Antibacterial Assessment

*Dipankar Ghosh, Sophie M. Coulter, Garry Laverty, Chris Holland, James Doutch, Massimo Vassalli, and Dave J. Adams**

SANS: Our group previously identified structural changes in 2NapFF wormlike micelles after adding calcium chloride.[1] Here, by comparing a set of nine gels, each cross-linked with a different metal, we aimed to elucidate the role of the linker in gel formation. The SANS data were fitted by combining a flexible elliptical cylinder and a hollow cylinder model (Table S1). Interestingly, 2NapFF is known to fit a hollow cylinder model at high pH (solution)[1] and a flexible elliptical cylinder at low pH (gel).[2]

Table S1. Fitting parameters for SANS data of 2NapFF-metal chloride bulk gels at 1:1 molar ratio

	2NapFF. Mg	2NapFF. Ca	2NapFF. Sr	2NapFF. Mn	2NapFF. Fe	2NapFF. Co	2NapFF. Ni	2NapFF. Cu	2NapFF. Zn
Model	Flexible elliptical cylinder + Hollow cylinder								
Background (cm^{-1})	0.025	0.018	0.02	0.01	0.03	0.015	0.015	0.01	0.02
A Scale	$1.40x10^{-4} \pm 1.53 x10^{-6}$	$4.64x10^{-4} \pm 5.68 x10^{-6}$	$1.23x10^{-4} \pm 1.36 x10^{-6}$	$1.81x10^{-4} \pm 2.83 x10^{-6}$	$3.45x10^{-4} \pm 5.08 x10^{-6}$	$1.44x10^{-4} \pm 3.71 x10^{-6}$	$1.29x10^{-4} \pm 1.44 x10^{-6}$	$3.91x10^{-4} \pm 5.23 x10^{-6}$	$1.96x10^{-4} \pm 3.64 x10^{-6}$
A Length (Å)	>1000	>1000	>1000	>1000	>1000	>1000	>1000	>1000	>1000
A Kuhn Length (Å)	257 ± 10	126 ± 3	231 ± 6	231 ± 12	177 ± 8	182 ± 26	395 ± 37	120 ± 5	471 ± 25
A Radius (Å)	77.9 ± 0.7	34.1 ± 0.3	62.8 ± 0.9	52.0 ± 1.4	27.8 ± 0.2	67.4 ± 1.1	70.2 ± 1.0	41.9 ± 0.4	50.9 ± 1.5
A Axis ratio	2.75 ± 0.03	2.03 ± 0.03	3.34 ± 0.05	1.96 ± 0.06	4.00 ± 0.06	1.93 ± 0.07	2.26 ± 0.05	1.47 ± 0.09	2.41 ± 0.07
B Scale	$1.56x10^{-3} \pm 2.51 x10^{-3}$	$3.94x10^{-4} \pm 7.09 x10^{-5}$	$1.45x10^{-3} \pm 2.07 x10^{-3}$	$5.34x10^{-4} \pm 4.54 x10^{-5}$	$5.76x10^{-4} \pm 1.02 x10^{-3}$	$1.23x10^{-3} \pm 1.87 x10^{-4}$	$1.45x10^{-3} \pm 3.61 x10^{-4}$	$1.78x10^{-4} \pm 7.00 x10^{-6}$	$8.61x10^{-4} \pm 7.31 x10^{-5}$
B Length (Å)	>1000	>1000	>1000	>1000	>1000	>1000	>1000	>1000	>1000
B Radius (Å)	24.2 ± 3.0	43.8 ± 1.5	24.2 ± 2.9	13.5 ± 1.1	47.0 ± 5.0	19.9 ± 0.8	21.7 ± 1.0	39.9 ± 1.8	17.0 ± 0.8
B Thickness (Å)	3.7 ± 6.0	13.7 ± 2.7	4.0 ± 5.8	16.3 ± 2.0	5.2 ± 9.7	9.2 ± 1.5	7.3 ± 1.9	29.0 ± 2.5	13.4 ± 1.8
χ^2	6.71	12.55	4.05	3.22	3.94	4.85	3.45	13.24	3.99

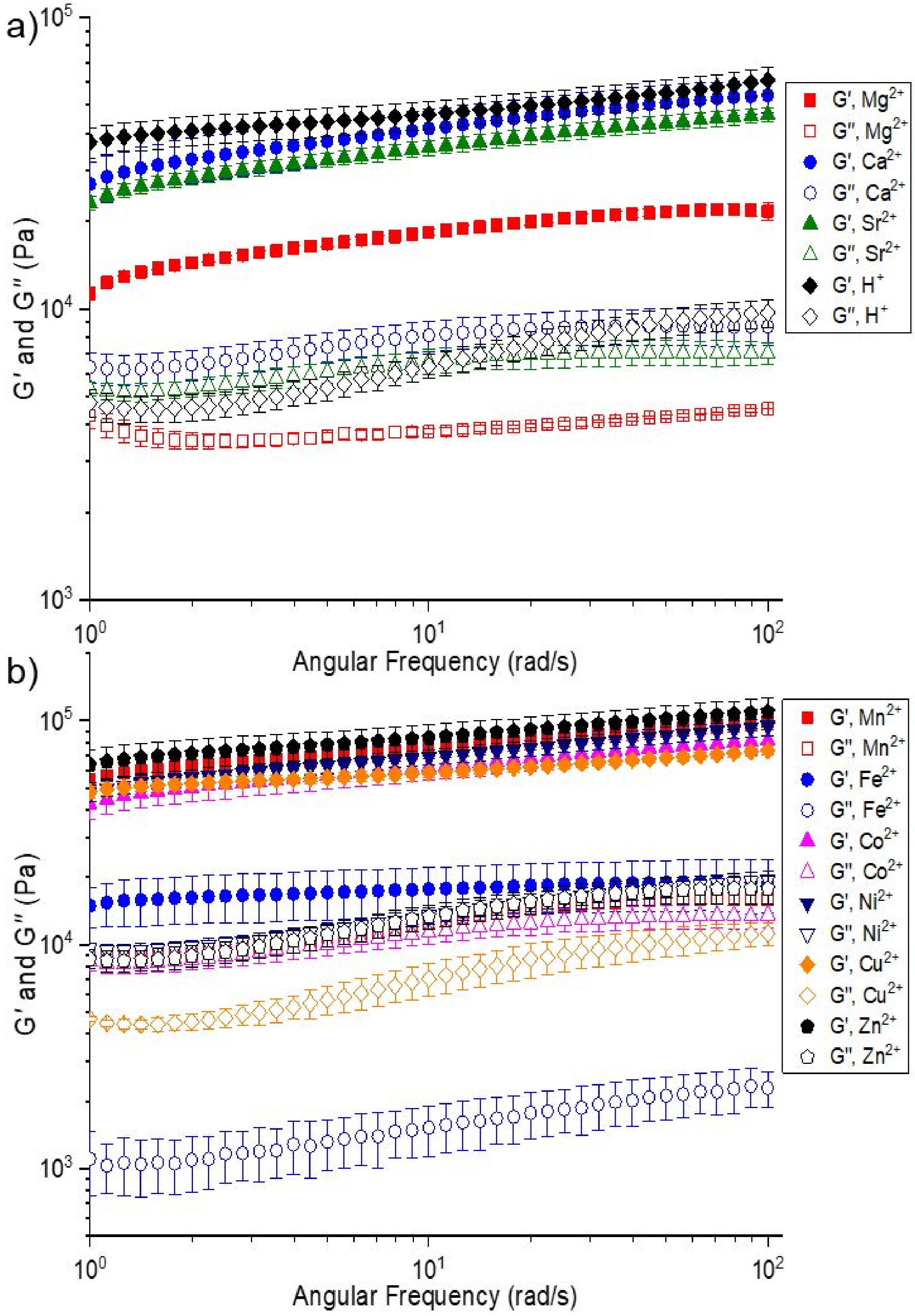

Figure S1. (a) Frequency sweep experiments on the gels obtained by mixing 1 mL 20 mg/mL, pH 10.5 2NapFF and 1 mL 40 mM Group 2 metal chlorides (or HCl). (d) Frequency sweep with *d*-block metal chlorides under the same condition.

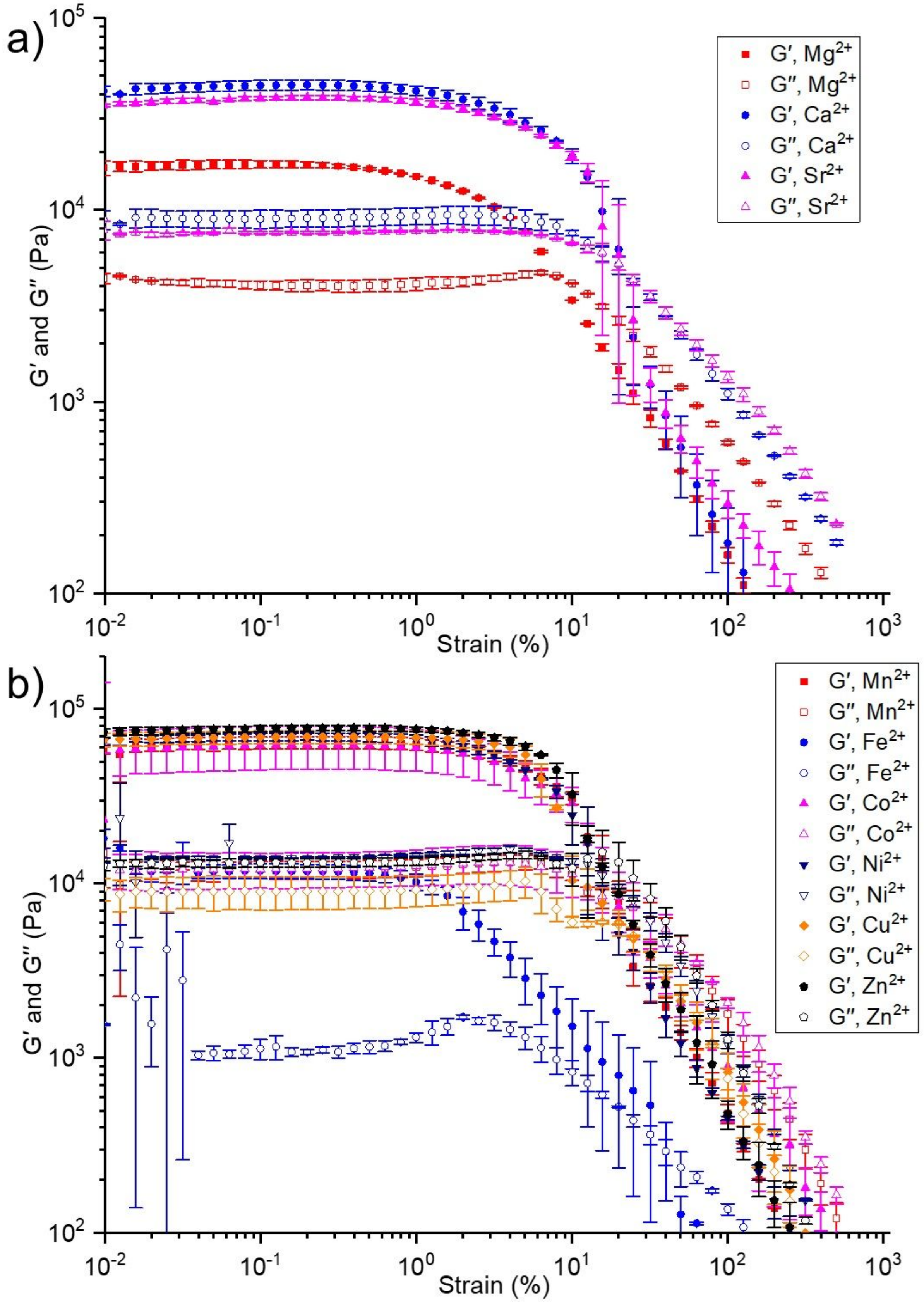

Figure S2. Oscillatory strain sweep experiments on the gels obtained by mixing 1 mL 20 mg/mL, pH 10.5 2NapFF and 1 mL 40 mM (a) Group 2 metal chlorides, (b) *d*-block metal chlorides.

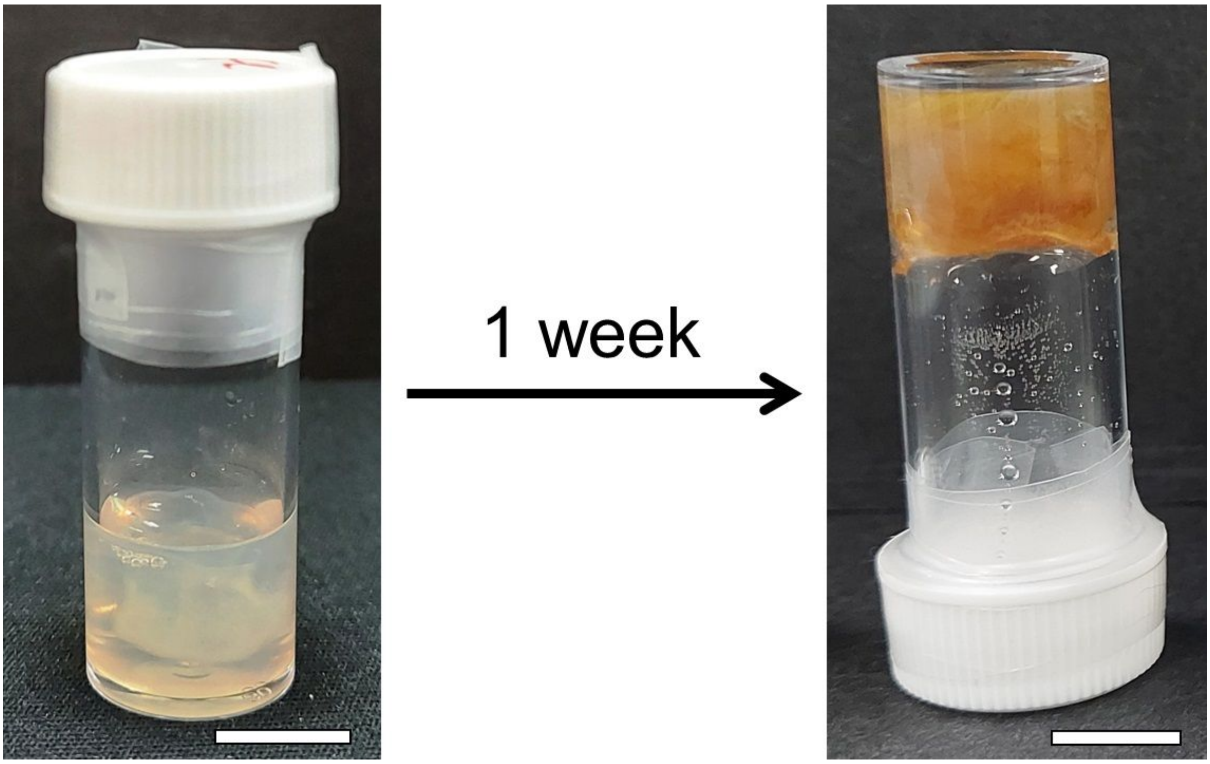

Figure S3. Bulk gel obtained by mixing 1 mL 2NapFF (20 mg/mL and pH 10.5) and 1 mL freshly prepared 40 mM $FeCl_2$, (left) immediately after mixing, and (right) after 1 week, the vial was inverted to confirm that the gel was stable. The color change strongly suggests the oxidation of Fe^{2+} to Fe^{3+}. The scale bars represent 1 cm.

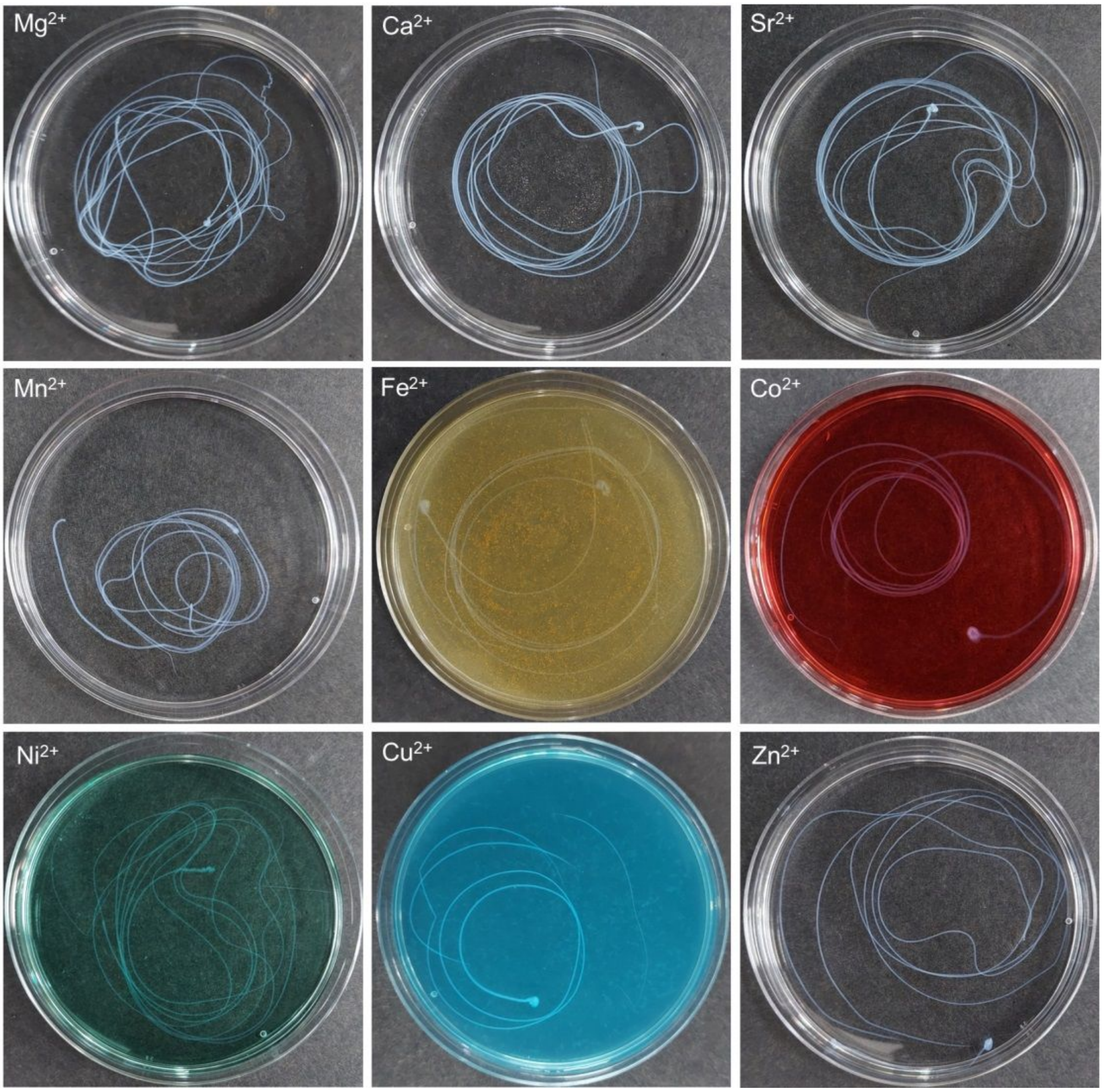

Figure S4. Gel noodles obtained with 2NapFF (concentration of 20 mg/mL and pH 10.5) and 0.5 M metal chlorides. The cations are indicated at the top left of each image. The diameter of the Petri dishes is 90 mm.

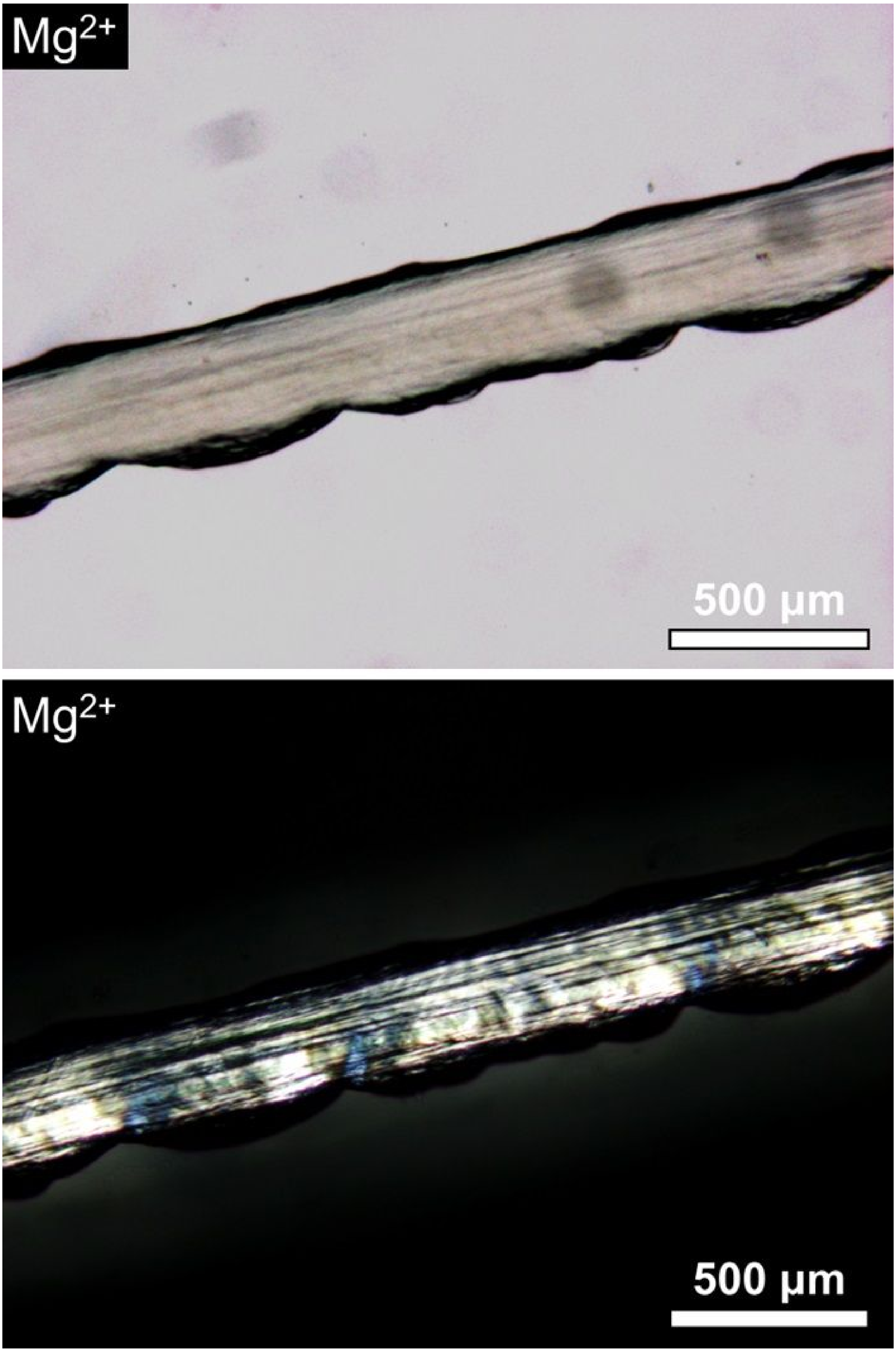

Figure S5. Microscopy images of the gel noodles obtained from 2NapFF and $MgCl_2$: (top) under visible light and (bottom) polarised light.

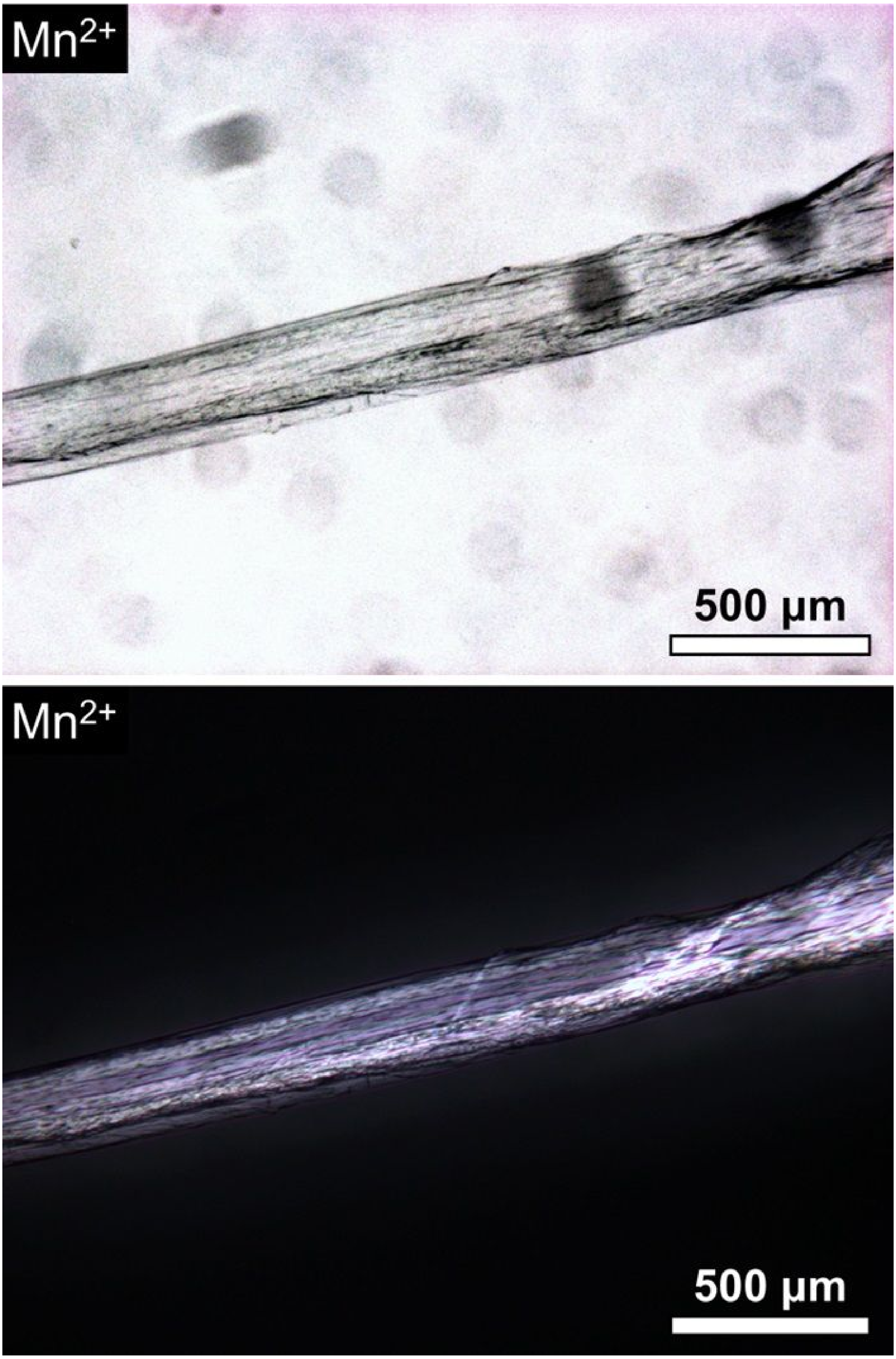

Figure S6. Microscopy images of the gel noodles obtained from 2NapFF and $MnCl_2$: (top) under visible light and (bottom) polarised light.

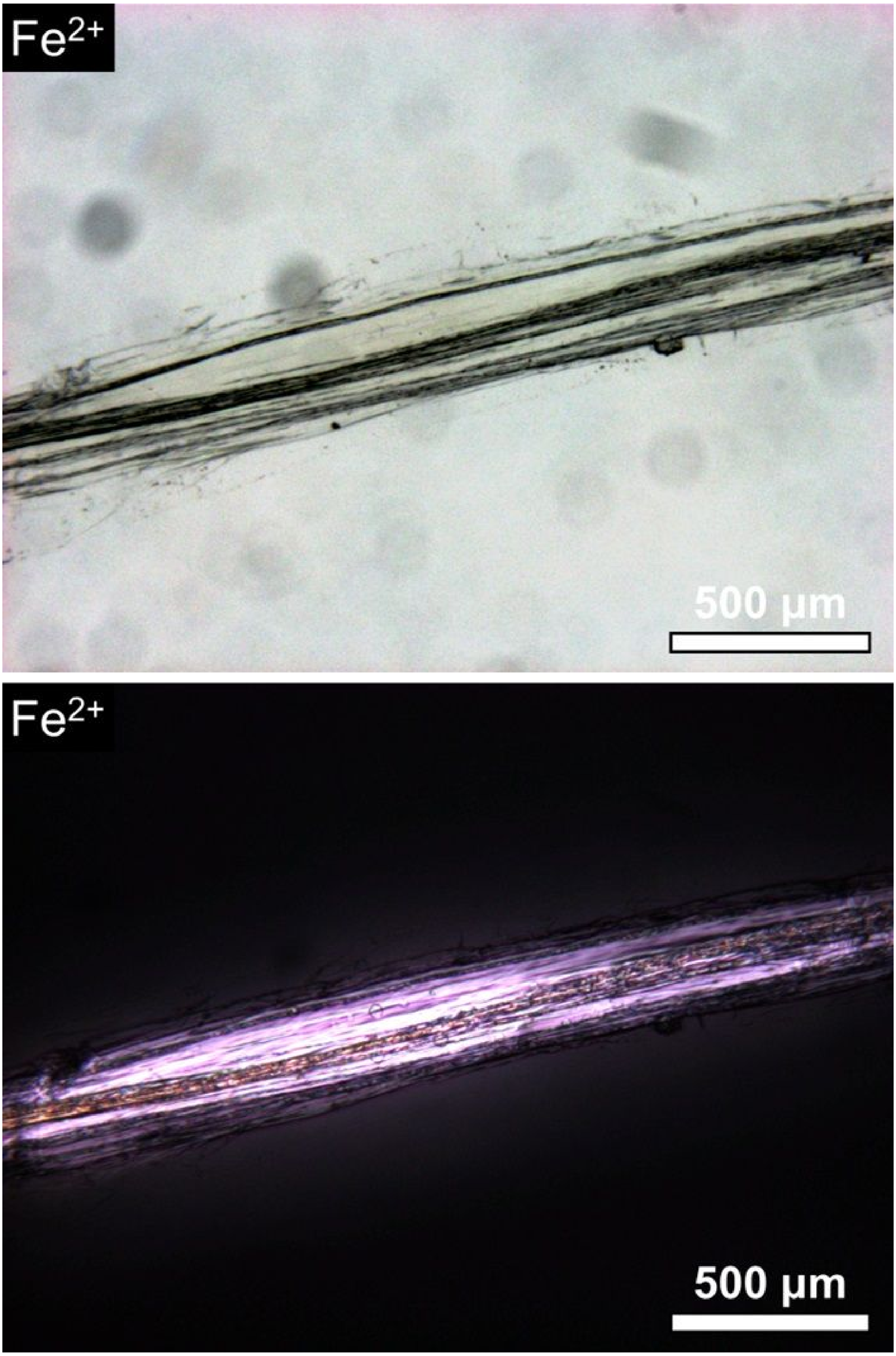

Figure S7. Microscopy images of the gel noodles obtained from 2NapFF and $FeCl_2$: (top) under visible light and (bottom) polarised light.

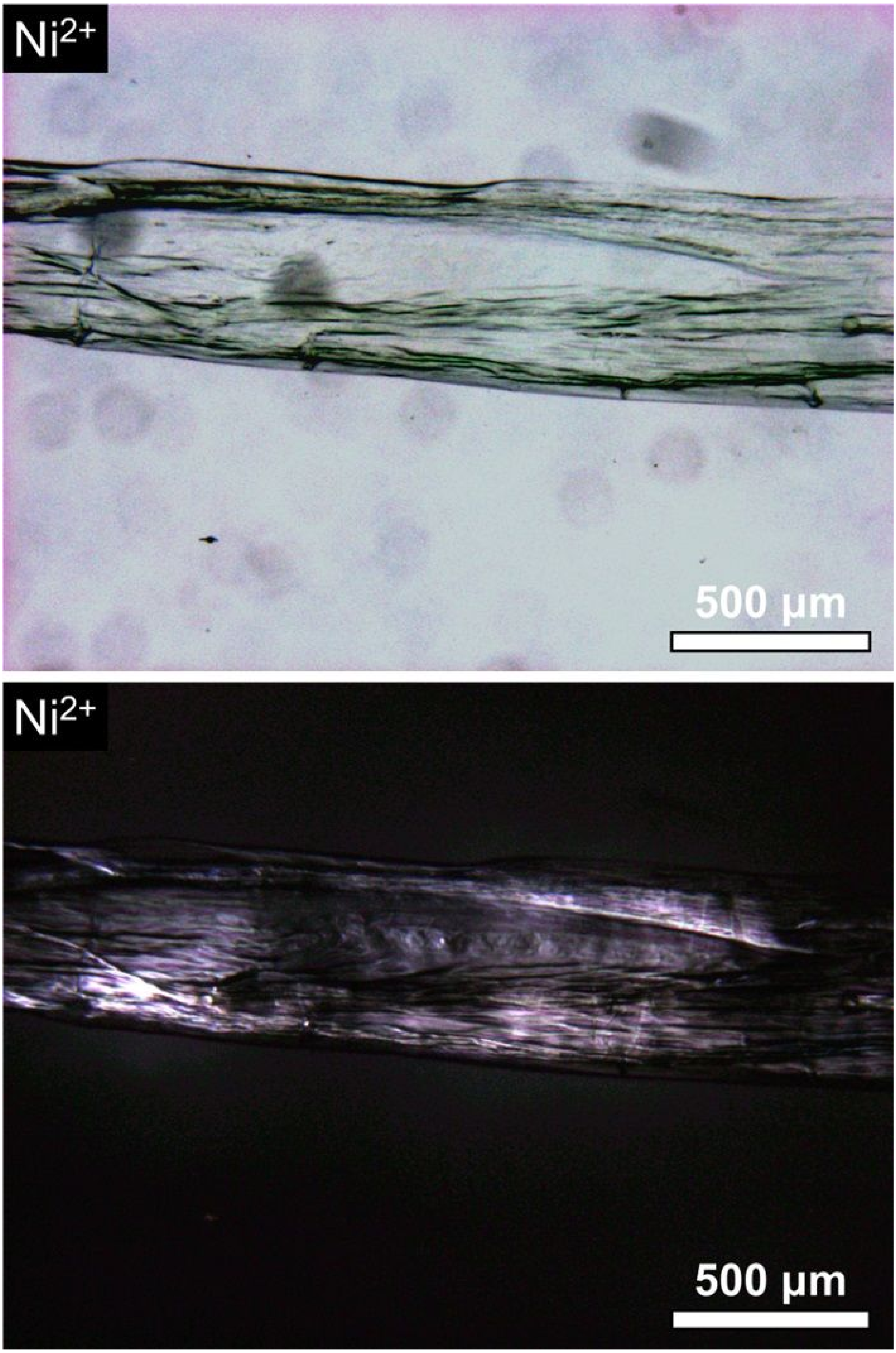

Figure S8. Microscopy images of the gel noodles obtained from 2NapFF and $NiCl_2$: (top) under visible light and (bottom) polarised light.

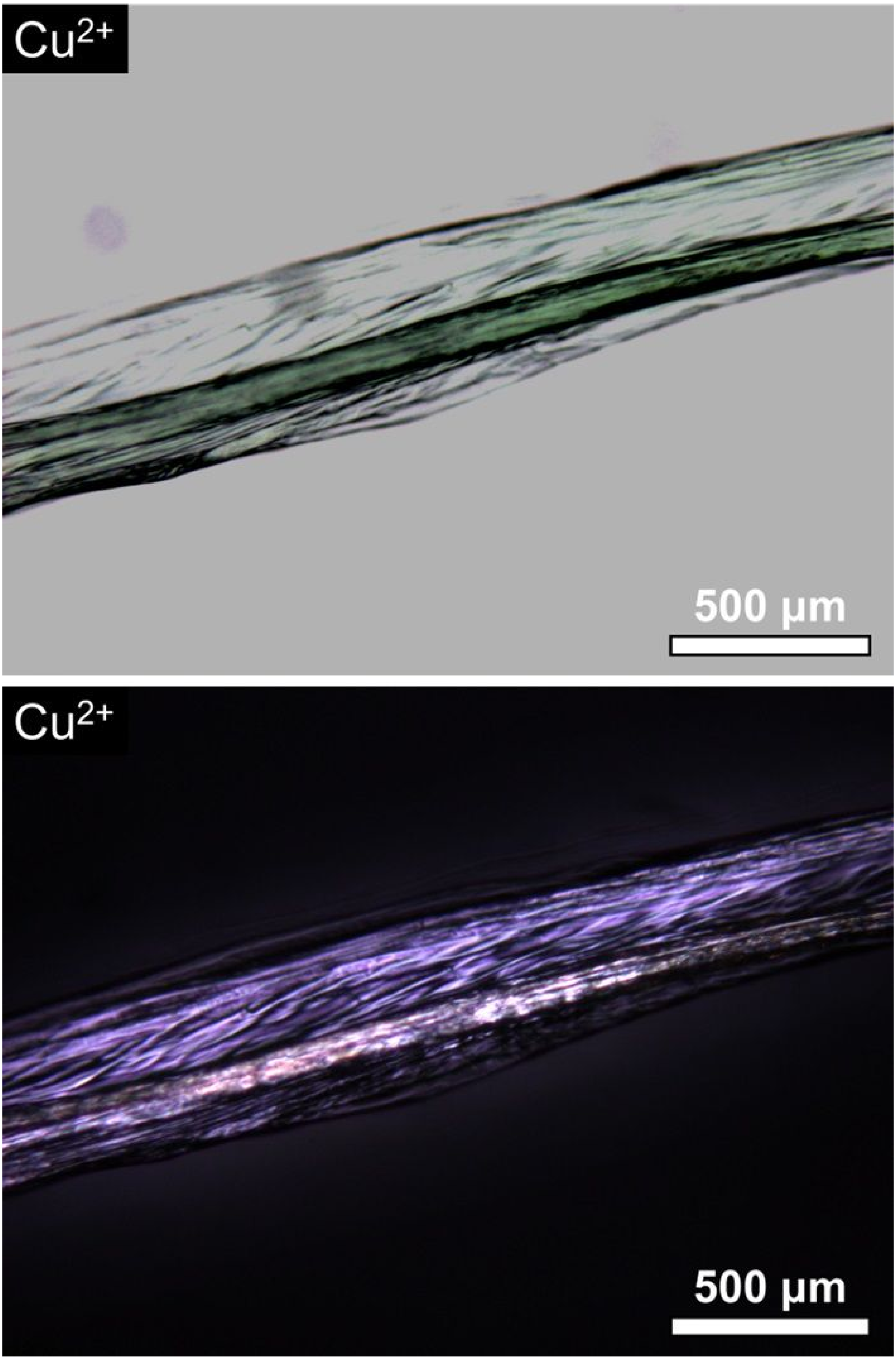

Figure S9. Microscopy images of the gel noodles obtained from 2NapFF and $CuCl_2$: (top) under visible light and (bottom) polarised light.

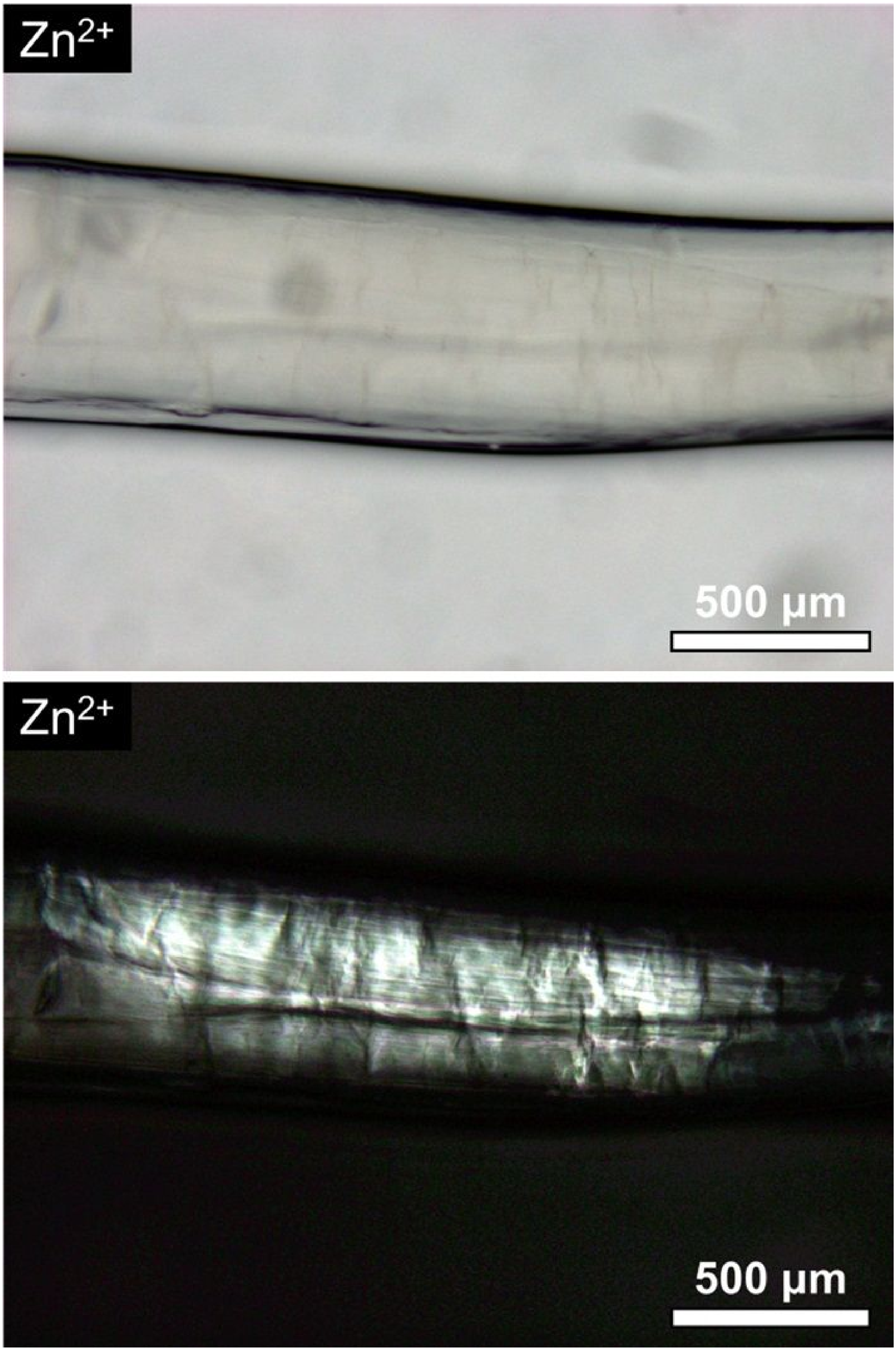

Figure S10. Microscopy images of the gel noodles obtained from 2NapFF and $ZnCl_2$: (top) under visible light and (bottom) polarised light.

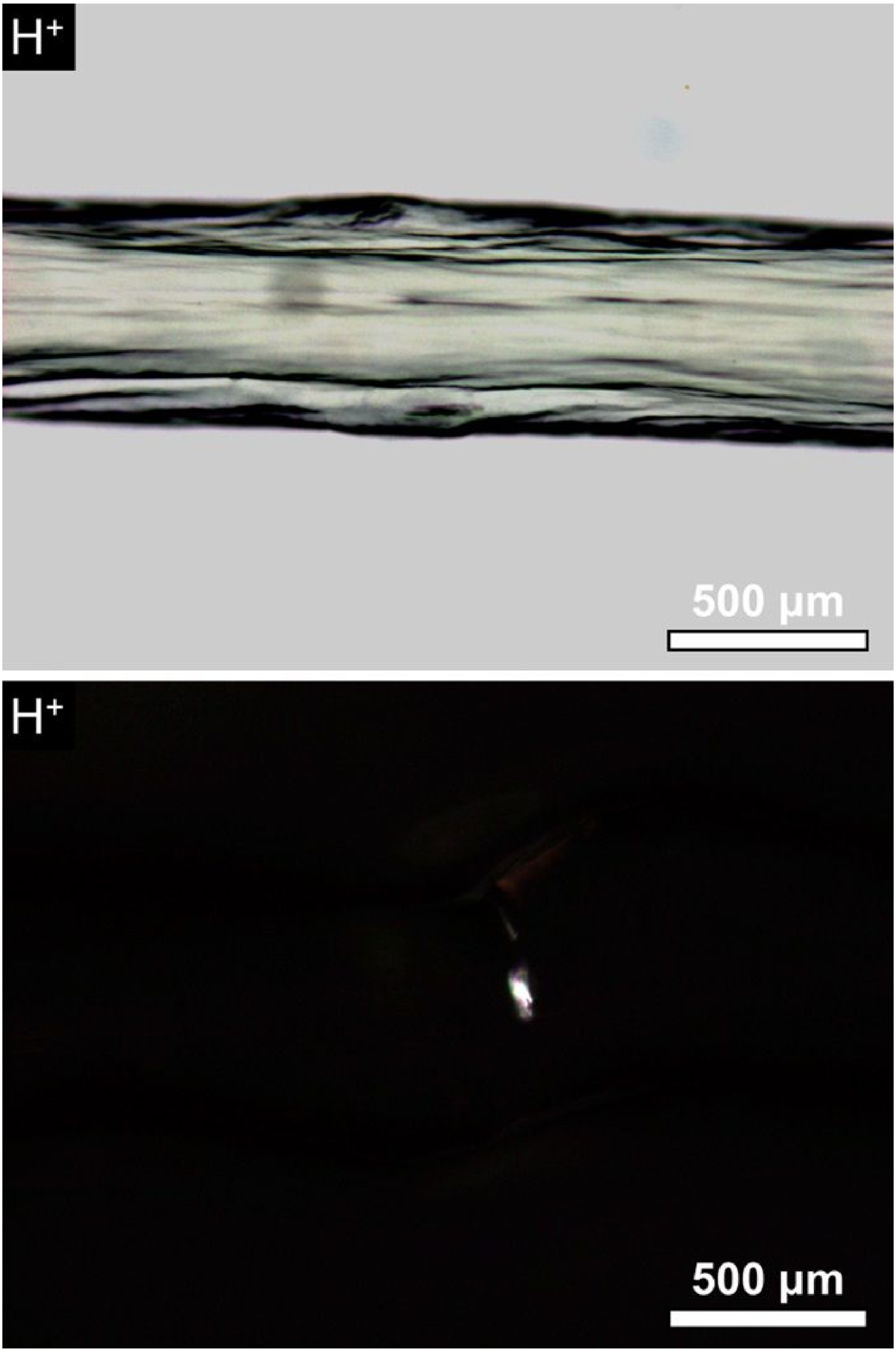

Figure S11. Microscopy images of the gel noodles obtained from 2NapFF and HCl: (top) under visible light and (bottom) polarised light.

Table S2: Average Young's Modulus (*E*) and standard deviation obtained from nanoindentation

Cross-linking ion	Young's Modulus (*E*) in kPa	Standard deviation (kPa)
Mg^{2+}	11.9	5.2
Ca^{2+}	15.3	9.7
Sr^{2+}	13.2	7.5
Mn^{2+}	12.8	7.5
Fe^{2+}	33.0	16.3
Co^{2+}	18.0	8.0
Ni^{2+}	15.6	9.8
Cu^{2+}	13.5	10.5
Zn^{2+}	11.5	6.2
H^{+}	26.0	12.6

Statistical analyses were performed using Microsoft Excel 2021 and GraphPad Prism 10.2.2. The Kruskal–Wallis test was used when the data were shown to be non-normally distributed according to the Kolmogorov and Smirnov test or standard deviations were shown to be statistically different using Brown-Forsythe and/or Bartlett's test. A Dunn's post-hoc test was used to identify individual differences between groups, for example the impact of using different cations on the Young's modulus. A probability of $p \leq 0.05$ denoted significance in all cases.

Table S3: Statistical analyses of the nanoindentation data

Dunn's multiple comparisons test	Mean rank diff.	Significant?	Summary	Adjusted *p* Value	
Mg vs. Ca	103.1	No	ns	0.5144	A-B
Mg vs. Sr	149.2	Yes	**	0.0076	A-C
Mg vs. Mn	164.4	Yes	**	0.0057	A-D
Mg vs. Fe	-257.3	Yes	****	<0.0001	A-E
Mg vs. Co	-16.11	No	ns	>0.9999	A-F
Mg vs. Ni	89.12	No	ns	0.9758	A-G
Mg vs. Cu	176.7	Yes	***	0.0002	A-H
Mg vs. Zn	200.9	Yes	***	0.0010	A-I
Mg vs. H	-169.9	Yes	***	0.0003	A-J
Ca vs. Sr	46.07	No	ns	>0.9999	B-C
Ca vs. Mn	61.29	No	ns	>0.9999	B-D

Ca vs. Fe	-360.4	Yes	****	<0.0001	B-E
Ca vs. Co	-119.2	No	ns	0.7202	B-F
Ca vs. Ni	-14.00	No	ns	>0.9999	B-G
Ca vs. Cu	73.56	No	ns	>0.9999	B-H
Ca vs. Zn	97.80	No	ns	>0.9999	B-I
Ca vs. H	-273.1	Yes	****	<0.0001	B-J
Sr vs. Mn	15.22	No	ns	>0.9999	C-D
Sr vs. Fe	-406.4	Yes	****	<0.0001	C-E
Sr vs. Co	-165.3	Yes	*	0.0302	C-F
Sr vs. Ni	-60.07	No	ns	>0.9999	C-G
Sr vs. Cu	27.49	No	ns	>0.9999	C-H
Sr vs. Zn	51.73	No	ns	>0.9999	C-I
Sr vs. H	-319.1	Yes	****	<0.0001	C-J
Mn vs. Fe	-421.7	Yes	****	<0.0001	D-E
Mn vs. Co	-180.5	Yes	*	0.0194	D-F
Mn vs. Ni	-75.29	No	ns	>0.9999	D-G
Mn vs. Cu	12.27	No	ns	>0.9999	D-H
Mn vs. Zn	36.51	No	ns	>0.9999	D-I
Mn vs. H	-334.3	Yes	****	<0.0001	D-J
Fe vs. Co	241.1	Yes	****	<0.0001	E-F
Fe vs. Ni	346.4	Yes	****	<0.0001	E-G
Fe vs. Cu	433.9	Yes	****	<0.0001	E-H
Fe vs. Zn	458.2	Yes	****	<0.0001	E-I
Fe vs. H	87.31	No	ns	>0.9999	E-J
Co vs. Ni	105.2	No	ns	>0.9999	F-G
Co vs. Cu	192.8	Yes	**	0.0024	F-H
Co vs. Zn	217.0	Yes	**	0.0035	F-I
Co vs. H	-153.8	No	ns	0.0508	F-J
Ni vs. Cu	87.55	No	ns	>0.9999	G-H
Ni vs. Zn	111.8	No	ns	0.8819	G-I
Ni vs. H	-259.1	Yes	****	<0.0001	G-J
Cu vs. Zn	24.24	No	ns	>0.9999	H-I
Cu vs. H	-346.6	Yes	****	<0.0001	H-J
Zn vs. H	-370.9	Yes	****	<0.0001	I-J

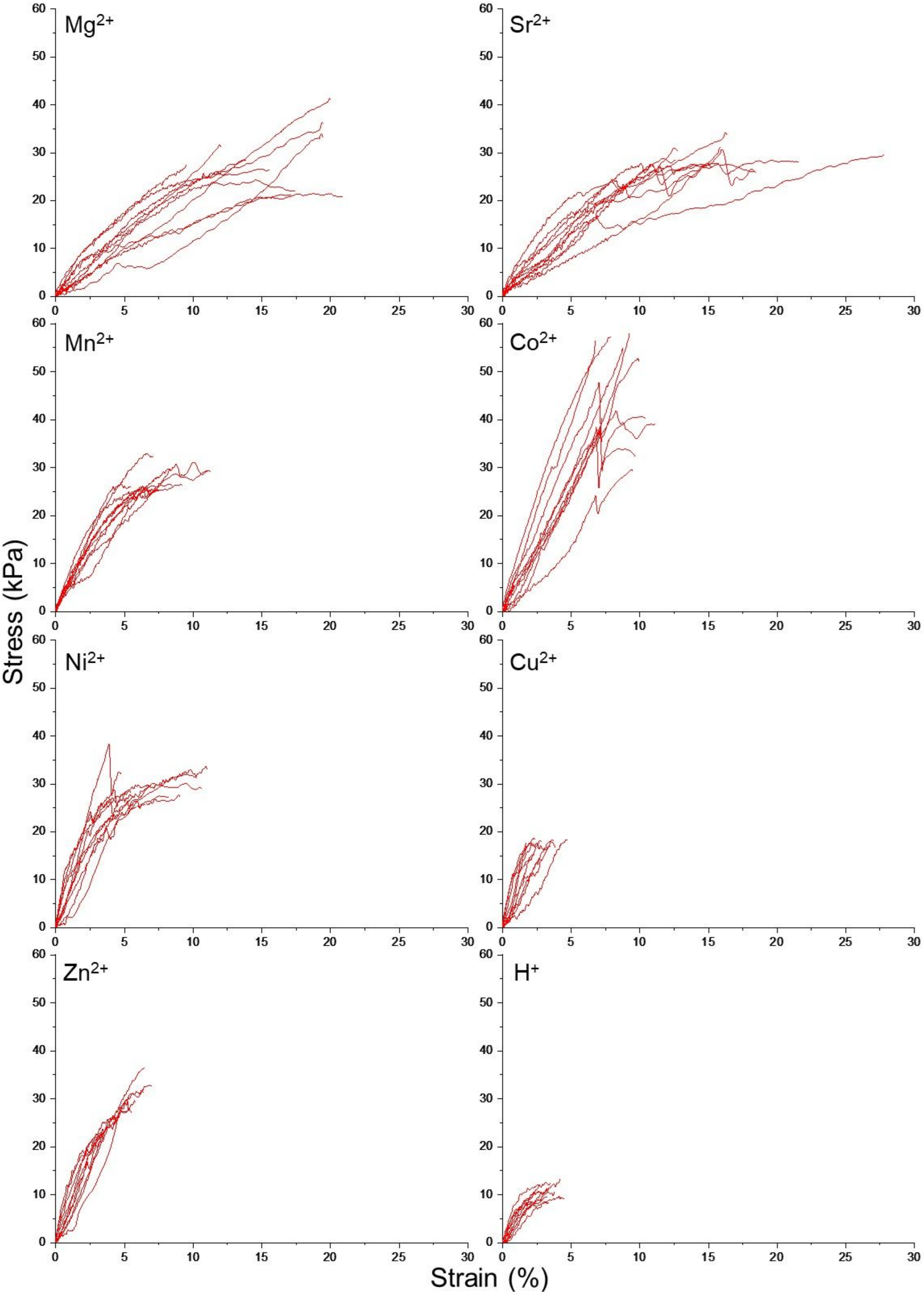

Figure S12. Tensile strength of 10 gel noodles obtained with 2NapFF and each metal chloride and HCl. The cations are indicated at the top left of each graph.

References:

1. Cardoso, A. Z.; Mears, L. L. E.; Cattoz, B. N.; Griffiths, P. C.; Schweins, R.; Adams, D. J., Linking micellar structures to hydrogelation for salt-triggered dipeptide gelators. *Soft Matter* **2016,** 12, (15), 3612-3621.
2. Draper, E. R.; Dietrich, B.; McAulay, K.; Brasnett, C.; Abdizadeh, H.; Patmanidis, I.; Marrink, S. J.; Su, H.; Cui, H.; Schweins, R.; Seddon, A.; Adams, D. J., Using Small-Angle Scattering and Contrast Matching to Understand Molecular Packing in Low Molecular Weight Gels. *Matter* **2020,** 2, (3), 764-778.
